# Management of Oral Feeding Challenges in Neonatal Intensive Care Units (NICUs): A National Survey in China

**DOI:** 10.3389/fped.2020.00336

**Published:** 2020-06-24

**Authors:** Tianchan Lyu, Yuxia Zhang, Xiaojing Hu, Ying Gu, Liling Li, Chantal Lau

**Affiliations:** ^1^Neonatal Intensive Care Unit, Children's Hospital of Fudan University, Shanghai, China; ^2^Nursing Department, Children's Hospital of Fudan University, Shanghai, China; ^3^Nursing Department, Zhongshan Hospital of Fudan University, Shanghai, China; ^4^Department of Pediatrics, Baylor College of Medicine, Houston, TX, United States

**Keywords:** high-risk infants, Chinese NICU, oral feeding practices, nursing management, nationwide survey

## Abstract

**Objectives:** To investigate the current practices of oral feeding difficulties facing high-risk infants in Chinese NICUs.

**Methods:** A questionnaire to survey infant oral feeding practices was distributed to 100 level II and III Chinese neonatal intensive care units (NICUs).

**Results:** Responses were obtained from 88 NICUs. No Units had any structured guidelines regarding the management of infant oral feeding as they transitioned from tube to independent oral feeding. In 54 (61.4%) NICUs, nurses and physicians made shared decisions on when oral feeding were to be initiated. Fifty-four (61.4%) and 22 (25.0%) NICUs used postmenstrual age (PMA) or weight at PMA as a criterion for initiating oral feedings, respectively. The top three criteria to determine introduction of oral feeding were severity of disease, presence of sucking reflex, and trial feeding success. Adverse events were used by 78 Units as indices of oral feeding difficulty. Twenty (22.7%) and 25 (28.4%) Units had access to occupational therapists or nurses who provided oral motor interventions during feeding, i.e., oral support (chin and cheek support, aid to deglutition), non-nutritive sucking with pacifier, and oral stimulation.

**Conclusions:** The management of oral feeding issues in NICUs vary widely in China in relation to the assessment of readiness to oral feeding, daily oral feeding practices and interventions used by staff. It is proposed that an educational program focused on the physiology of infant oral feeding, available evidence-based tools and interventions would assist NICU caregivers develop structured guidelines to improve infants' safe and efficient attainment of independent oral feeding.

## Introduction

As a result of medical technology and advances in perinatal care, the survival of preterm infants has increased in the last decade in China (1). After infants in neonatal intensive care units (NICUs) overcome life-threatening morbidities and chronic conditions associated with prematurity, their hospital discharge is often delayed because of their inability to feed safely and efficiently by mouth (2, 3). The attainment of independent oral feeding is one of the discharge criteria as oral feeding difficulties during infancy can lead to long-term feeding problems and can be detrimental to the quality of life of these infants and their families (4, 5). Consequently, the difficulties encountered by these infants' ability to readily feed by mouth have been gaining attention in recent years in China as it has in other countries.

Previous studies have indicated that differences in nursery management practices may impact an infant's oral feeding outcome. Several scales have been developed to assess the ability of neonates to begin suckle feeding and assist caregivers in determining feeding advancement (6–11). However, feeding preterm neonates remains an ongoing challenge and depends on the caregivers' feeding expertise. Some studies have shown that a nurse-based oral feeding protocol or program has a positive impact on the achievement of full oral feeding in these infants (12–14). However, discussions within NICUs in China have suggested that few hospitals have oral feeding protocols/guidelines resulting in large variations in practice. These differences are reflected in the varied indicators used to assess infants' readiness to start oral feeding, and the nature of their oral feeding difficulties. Meanwhile, as most Chinese NICUs do not allow parents to visit their infant(s), there is limited opportunity for breastfeeding and mother-infant nurturing and bonding (15). To the best of our knowledge, this is the first nationwide prospective survey conducted on the oral feeding management of high-risk infants in Chinese NICUs.

## Methods

This study was conducted in NICUs with the cooperation of Subspecialty Group of Nursing, Society of Pediatrics, and Chinese Medical Association. The survey was administered between September and November 2018 using a link to a web-based survey program (SoJump, Ranxing, China) via a WeChat group that includes all members of these three organizations. As most preterm neonates commence oral feeding during their NICU stay, questionnaires were sent to each Unit head nurse listed in the organization. Every head nurse received an electronic cover letter explaining the purpose of the study, a questionnaire, and the survey link. This was followed by a reminder email a month later to gain maximum participation. The participants from whom consent was obtained were informed that the results of the study would be published in a form that did not allow for the identification of their individual nursery.

The questionnaire items were generated through a review of the literature related to the practice of oral feeding in their respective neonatal wards (4, 9, 16, 17). Three experienced nurses and two physicians who worked in neonatal wards for more than 5 years tested the survey for content validity. They provided feedback on clarity, ease of understanding, formatting, and length of time to complete the questionnaire. Five nurses who piloted the survey were not associated with the development of the questionnaire and were excluded from the survey responses.

The questionnaire consisted of three sections. In the first section, head nurses were asked to fill out the questionnaire regarding their hospital characteristics, such as hospital location, NICU levels, visitor policy. The second section focused on head nurses' professional background, i.e., years of experience and professional titles. The third section was used to obtain information on the nursing management practice of oral feeding issues within their NICU, including nursing attitudes toward the current oral feeding practice and the conditions that facilitate/hinder advancement of oral feeding. Specifically, head nurses were also asked to provide the following information: (a) criteria, e.g., gestational age, weight, assessments/checklists or guidelines used to determine the readiness to oral feeding, (b) daily oral feeding practice including the choices of oral feeding, e.g., breast- or bottle-feeding, the oral feeding progression, e.g., number of oral feedings/day, the feeding tolerance assessment, e.g., gastric residuals evaluation, the record of oral feeding amount per day or not and the pacifier use or not, (c) criteria, e.g., post-menstrual age, episodes of adverse event, used to define the oral feeding difficulties and (d) specific interventions, e.g., non-nutritive sucking using pacifier, oral feeding support (chin and cheek support) during feeding, and oral stimulation (stroking perioral and intraoral structures) used to assist in their respective oral feeding practices. All questions in the survey were multiple-choice.

## Results

### Demographics

**Figure 1:**
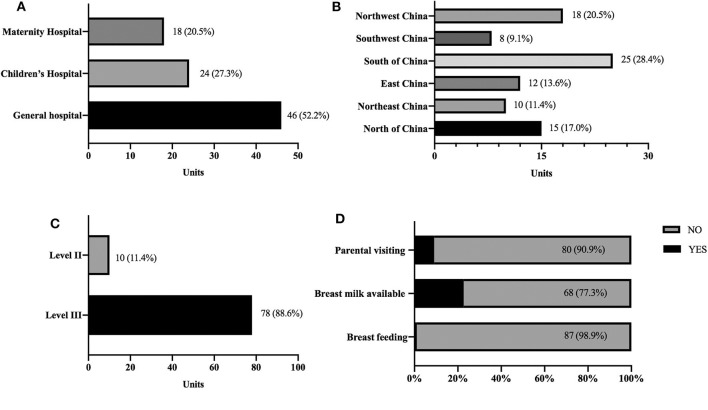
Demographics of the 88 participating NICUs; **(A)** Hospital type; **(B)** Hospital location; **(C)** Classification of NICU; **(D)** Family-centered care policy; Numbers (%) of Units for each category are listed on each graph.

We identified 100 Units providing neonatal intensive care in the hospitals surveyed within the Subspecialty Group of Nursing, Society of Pediatrics of the Chinese Medical Association. Of these 100 Units, 88 (88.0%) responded to the questionnaire. Among the Respondent head nurses, 48 (54.5%) and 40 (45.5%) had more than five and 10 years of experience as neonatal nurses, respectively. The characteristics of the participating NICUs are shown in Figure 1. Eighteen (20.5%) units were part of maternity hospital, 24 (27.3%) from Children's hospital, and 46 (52.2%) from General hospitals (Figure 1A). Participating NICUs were distributed evenly throughout China (Figure 1B). Seventy-eight (88.6%) of the surveyed participants identified their site as level III NICUs, with infants born <32 weeks gestational age (GA) and high-risk infants of all GAs. Ten (11.4%) NICUs provided level II care for infants born >32 weeks GA or transferred from level III (Figure 1C). Parents were not allowed to visit their infants during their infants' hospitalization in 80 (90.9%) NICUs. In the eight Units that allowed parental visits, the frequency of visits ranged from once a day to once every 2 weeks. In 68 (77.3%) Units, the infants had no access to mother' own milk when bottle feeding, while only one Unit allowed breastfeeding during parental visits (Figure 1D). Median number of NICU beds among respondents was 56 (range 20–120); that of annual newborns admission was 580 (range 80–1,200), VLBW admission was 20 per year (range 10 to 450). The range for gestational age and birth weight was 24 to 31 weeks GA and 560 to 1,700 g, respectively.

### Hospitals That Provide Oral Feeding Management Care

Eighty-five (97.0%) NICUs had no staff responsible for infant oral feeding advancement. Three (3.4%) NICUs had internationally board-certified lactation consultants (IBCLCs) in charge of oral feeding assessment. In 54 (61.4%) NICUs with 44 level III and 10 level II Units, the nurses and physicians together decided on when to initiate oral feeding. In 16 (18.2%) level III Units, physicians alone were in charge of such decision whereas in 15 (17.4%) level III Units only nurses were in charge of managing oral feeding progress. In only two NICUs, nurses made the decision of removal of gastric tube.

### Criteria for Oral Feeding Initiation

**Figure 2:**
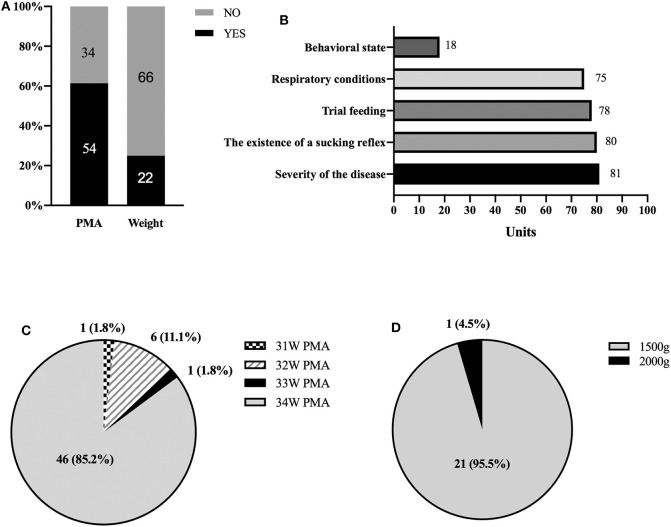
Criteria used to determine the initiation of oral feeding; **(A)** PMA and weight; **(B)** other criteria other than PMA and weight; **(C)** Among the Units relying on PMA, number (%) of Units cared for by PMA; **(D)** Among the Units relying on infant weight, number (%) of Units cared for by birth weight.

None of the participating NICU reported having any formal, written policies or protocols regarding the management of oral feeding issues in preterm infants. Fifty-four (61.4%) and 22 (25.0%) NICUs relied on the postmenstrual age (PMA) or weight at PMA as the criterion for initiating oral feedings, respectively (Figure 2A). Only eight NICUs relied on both PMA and infant weight at PMA. Other criteria used to determine the initiation of oral feeding outlined included severity of the disease (defined by the Neonatal Medical Index, NMI) (18), presence of a sucking reflex (by finger or cotton swab), trial feeding, receiving respiratory condition or not, and behavioral state (defined by Anderson Behavioral state scale) (19) (Figure 2B); Among the 54 Units relying on PMA, 46 reported 34 weeks PMA as the minimum age to start oral feedings while 31~33 weeks PMA were reported by others (Figure 2C). In the 22 Units that relied on infant weight, 21 NICUs reported 1,500 grams as minimal weight for introduction of oral feeding, while only one Unit reported 2,000 g (Figure 2D).

### Daily Oral Feeding Practice

The daily clinical practice of feeding premature infants varies between hospitals. Of the 88 NICUs, 87 (98.8%) used bottle feeding at the first oral feeding; only one hospital implemented breastfeeding. The daily feeding process of “oral feeding followed by tube feeding in every feeding” is utilized by 85 (96.5%) of 88 Units. Only 3 Units chose to begin 1 or 2 oral feeding in the first day with advancement of oral feeding/day based on infant weight gain and nursing evaluation of their oral feeding performance. Moreover, 70 (79.6%) NICUs recorded the amount of individual infants' oral feeding in detail on the nursing chart. Seventeen (19.3%) Units did not record the amount of each oral feeding in infants' medical records, but did so only verbally. Only one hospital mentioned that it did not pay attention to the amount taken at each feeding. Sixty (75.0%) Units gave pacifiers to infants before tube feeding, only 18 (20.5%) Units provided pacifiers before oral feeding. Eighty-eight (100%) Units evaluated feeding tolerance (gastric residual, episodes of vomiting, regurgitation) in every infant.

### Definition and Treatment of Oral Feeding Difficulty

Seventy-seven NICUs (87.5%) were aware of the occurrence of oral feeding issues. The hospital discharge criteria of all NICUs included attainment of full oral feeding. Of the 88 Respondents, 74 (84.1%) had infants with delayed discharge because they could not achieve full oral feeding.

**Figure 3:**
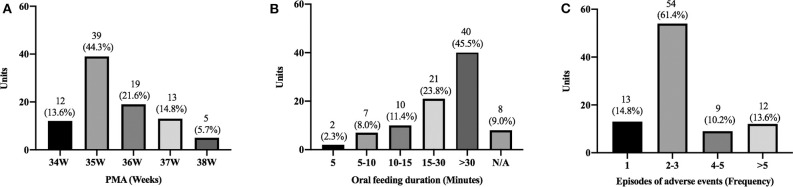
Number of Units (Y-axis) using the following criterion to define oral feeding difficulties: PMA **(A)**, duration of oral feeding **(B)**, and number of adverse events **(C)**.

However, the criteria to define or determine the oral feeding difficulties varied among the respondent Units. For instance, 39 (44.3%) Units considered oral feeding difficulties present if an infant did not achieve full oral feeding by 35 weeks PMA (Figure 3A). Forty (45.5%) Units considered oral feeding difficulty present if a single feeding exceeded 30 min. Eight (9.0%) Units did not consider oral feeding duration as a criterion to determine oral feeding difficulties (Figure 3B). Fifty-four (61.4%) Units used adverse events as a criterion if 2–3 adverse events occurred during a feeding, e.g., oxygen desaturation, asphyxia, bradycardia (Figure 3C).

**Figure 4:**
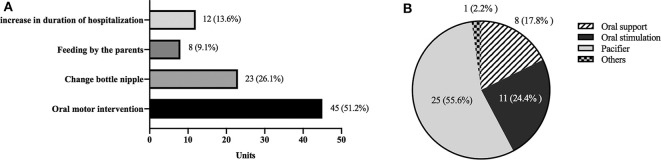
Treatment of oral feeding difficulties by: **(A)** Categories of oral feeding difficulty treatment, **(B)** Number (%) of differing oral motor interventions used by 45 Units.

For treatment of oral feeding difficulty (Figure 4A), 12 (13.6%) Units had no solution but continued tube feeding which prolonged hospital stay; eight (9.1%) Units involved parents in the oral feeding process presuming that parent-infant bonding would facilitate the oral feeding; 23 (26.1%) Units preferred changing bottle nipples based on size of nipple hole or texture; 45 (51.2%) Units used oral motor interventions. Twenty (22.7%) and 25 (28.5%) Units had access to occupational therapists and nurses who offered oral motor interventions, respectively. Figure 4B shows that among these 45 units cheek/chin support was used in eight (17.8%) units, oral stimulation and pacifiers was used in 11 (24.4%) and 25 (25.6%), respectively. Only one unit reported using tactile, olfactory, and visual stimulations from the literature (20) (Figure 4B).

### Staff Attitude Toward Infant Oral Feeding Challenges

Eighty-five respondents (97.7%) deemed that early assessment of oral feeding performance was important for the identification of high-risk infants and that early intervention should be offered. Listed obstacles included lack of reliable assessment tools (84.1%), lack of knowledge of available evidence-based interventions (68.2%) and attention by the medical staff (62.5%), overloaded clinical responsibilities (68.2%). Availability of easy-to-use assessment tools (69.3%) was recognized as being of great help to clinical practice.

## Discussion

This report conducted with the collaboration of the Subspecialty Group of Nursing, Society of Pediatrics, and Chinese Medical Association is the first survey, to our knowledge, that documents the medical practices and staff attitudes toward infant oral feeding challenges in NICUs throughout China. It describes the wide variations in practices of neonatal wards regarding the management of oral feeding issues as preterm neonates transition from tube to independent oral feeding. From the feedback received in this survey from NICU head nurses, the following observations may be summarized as follow: (1) there is the recognition that proper oral feeding management ought to be a primary focus of the neonatal staff caring for NICU infants, feeding strategies should be under continued review as oral feeding success is the infant milestone that often opens the door to NICU discharge (21). (2) Our current findings are consistent with results from international studies (16, 17) showing no evidence-based objective approaches to guide infants' transition to independent oral feeding. (3) As several devices/scales or interventions have been identified to help infants be successful with oral feeding (22–25), it is proposed that offering a systematic educational/training program to introduce the current available evidence-based techniques to neonatal care providers would greatly advance the care we can offer to our NICU babies in China.

In the absence of a uniform structured guideline, individual units have developed their own approach based on a wide range of subjective criteria. Caregivers predominantly use “trial-and-error” approaches, infants' GA and/or weight to determine oral feeding readiness. The decision to introduce and advance oral feeding lies primarily on the attending physicians and/or nurses. If an IBCLC is available, he/she would take on such responsibility. It is proposed that an improved approach to assist these infants' transition safely and efficiently from tube to independent oral feeding ought to rely on the multidisciplinary team members caring for them, i.e., physicians, nurses, lactation consultants, feeding therapists, insofar as their respective expertise and interactive inputs would significantly impact on the overall management of oral feeding performance of their *individual* infants. If “mother's milk is best”, then family involvement in their care early on in the NICU would be optimal. Indeed, the latter may help alleviate maternal stress, thereby safeguarding her interest in breastfeeding and/or pumping in order to continue providing her own milk to her infant (26). Studies have demonstrated that preterm infants fed directly at the breast receive human milk for longer period of time than those who receive expressed human milk from a bottle (27, 28). However, in our survey only one NICU chose breastfeeding over bottle feeding. Although bottle feeding may be a necessity under certa*in situ*ations, breastfeeding should be encouraged when it is consistent with the mother's goal (29), not only for the nutritional value of mother's milk, but importantly for the nurturance infants receive from their mother during breastfeeding (26). Unfortunately, in China, the biggest obstacle for breastfeeding in NICUs is the limited space around the incubators which restricts parental visits and involvement in their infant care. Fortunately, the family-centered care approach is gaining momentum among Chinese NICU clinicians (15) and changes of visit policy should be expected in the near future.

A number of management plans, oral feeding skills assessment program and scales, efficacious tools, and interventions have been developed, but their awareness has not been widely recognized and adopted into practice (10, 22–24, 30–33). Insofar as not all the respondents in our survey used oral motor interventions, the development of a training program that incorporates the current available evidence-based techniques to NICU providers would further advance the care we can offer our high-risk infants in China. Infant Driven feeding may be a potential approach to support these infants (21, 25). However, as per a recent Cochrane Review, its validation and acceptance will require more evidence-based and stringent studies (34). Currently, it is not an approach that is recognized in China.

In our survey, no protocol was available as to how best to advance oral feedings. Among the Respondents, once oral feeding was initiated, it was offered at every feeding to increase oral feeding opportunities. Further studies are needed to define what an optimal oral feeding advancement protocol should consist of. In this regard, the availability of the Oral Feeding Skills (OFS) scale may be of value as it can objectively monitor infants' oral feeding ability based on their endurance and nutritive sucking skills at any feeding without the need for any special device, but solely an additional measure of milk intake at a particular time during a feeding session (32). Such data recorded onto infants' clinical chart would provide an objective temporal profile of the maturation of these infants' oral feeding skills.

Inasmuch as attainment of full oral feeding is one of the criteria for NICU discharge (3), identification and intervention of oral feeding difficulties becomes germane. Previous studies have shown that the OFS scale and the “Infant Driven Scale” (24) may allow for early identification of infants at risk for delayed attainment of oral feeding independence. Adoption of such tools may help tailor appropriate interventions to support infants with oral feeding difficulty, but from our survey, no such tools have yet been recognized.

The results of this survey represent the current practice of infant oral feeding in Chinese NICUs. Although the results were based on feedback from NICUs head nurses who may not have had the hands-on experience of their staff, they offer an insight to the current oral feeding practices within their respective discipline.

In brief, as we know, this study is the first that has been conducted to identify the knowledge-based of NICUs healthcare providers in China as the increased survival of preterm infants and the growing awareness of their oral feeding challenges has become a public health concern. It is reported that 20–50% of healthy developing children encounter oral feeding difficulties and the incidence can rise to 80% for children with developmental disabilities and complex medical conditions, e.g., prematurity, cerebral palsy (35). The estimated preterm birth rates in China was 7.8% with 1.17 million livebirths in 2014 (36). If 80% of these high-risk infants have oral feeding difficulties, 0.93 million infants/year would be expected to face oral feeding challenges. As individual NICU nurses care for ≥5 infants in our NICUs, it becomes difficult to establish a comprehensive oral feeding program without the collaboration of team members, physician, feeding therapists, lactation consultants, and, importantly, parents.

## Conclusion

With the increased survival of premature babies, medical staff are increasingly aware of the oral feeding issues they encounter. This survey is a first that describes the broad variations in the clinical management of these infants as it relates to oral feeding initiation assessment, daily oral feeding practices and interventions used by staff. Further research and educational programs are needed to establish appropriate guidelines to promote oral feeding for these infants, assist caregivers rationally understand the causes of their difficulties and identify effective evaluation tools. Understanding the neuro-motor development of infant oral feeding skills, awareness of available evidence-based instruments would be a reasonable start in improving the clinical practice for these young patients' care as their hospitalization would be shortened, resulting in decreased medical expenses, and maternal stress along with earlier family reunification.

## Data Availability Statement

The datasets presented in this article are not readily available because they contain information that could compromise the research participants' privacy/consent. Requests to access the datasets should be directed to Tianchan Lyu, lvtianchan1988@aliyun.com.

## Ethics Statement

The studies involving human participants were reviewed and approved by ethical committee of Chinldren's Hospital Of Fudan University. The participants provided written informed consent to participate in this study.

## Author Contributions

YZ and TL contributed to the conception and design of the study. LL and YG organized the database. XH performed the statistical analysis. TL wrote the first draft of the manuscript. CL revised the manuscript critically. All authors contributed to manuscript revision, read and approved the submitted version.

## Conflict of Interest

The authors declare that the research was conducted in the absence of any commercial or financial relationships that could be construed as a potential conflict of interest.
